# Molecular analysis of appendiceal mucinous cystadenoma and rectal adenocarcinoma in a patient with urothelial carcinoma: a case report

**DOI:** 10.1186/1752-1947-7-170

**Published:** 2013-06-28

**Authors:** Fabio Pulighe, Panagiotis Paliogiannis, Antonio Cossu, Giuseppe Palmieri, Maria Colombino, Fabrizio Scognamillo, Mario Trignano

**Affiliations:** 1 Department of Surgical, Microsurgical and Medical Sciences, University of Sassari, Viale San Pietro 43B, 07100 Sassari, Italy; 2 Institute of Biomolecular Chemistry, Cancer Genetics Unit, C.N.R., Traversa La Crucca 3, 07040 Sassari, Italy

**Keywords:** Appendix, Colorectal cancer, Cystadenoma, Mucocele, Urothelial carcinoma

## Abstract

**Introduction:**

In this report, we present the case of a patient affected by appendiceal cystadenoma, a colorectal adenocarcinoma, and a concomitant bladder carcinoma, as well as the results of the molecular study of the most relevant mutational pathways involved in these tumors.

**Case presentation:**

A 68-year-old Italian man was admitted to our unit complaining of macrohematuria, rectorrhagia, and rectal tenesmus for about 2 months. A colonoscopy showed the presence of a rectal lesion at 11cm from the anal margin; multiple biopsies were performed and a diagnosis of moderately differentiated adenocarcinoma was made. Abdominal ultrasonography and total body computed tomography performed subsequently to stage the rectal cancer showed the presence of two round nodules, interpreted as swollen lymph nodes of neoplastic origin, at the anterior aspect of the iliopsoas muscle and a budding lesion affecting the bladder. The patient underwent transurethral biopsy of the lesion in the right retrotrigonal region; the diagnosis was grade II urothelial carcinoma. The patient underwent an open anterior rectal resection with loco-regional lymphadenectomy. An enlarged appendix and a voluminous whitish soft-tissue lesion requiring an appendicectomy were detected perioperatively. Transurethral resection of the bladder lesion was also performed. The histological examination revealed that the nodular lesions in the appendix were due to a cystadenoma. For mutation analysis, genomic deoxyribonucleic acid was isolated from tumor tissue samples; for *PIK3CA* mutations, screening revealed that all three samples analyzed carried mutations in exon 9.

**Conclusions:**

Appendiceal mucoceles are rare but require adequate surgical treatment, given their malignant potential and the possibility of causing peritoneal pseudomyxoma. It is essential to make a correct preoperative evaluation based on a colonoscopy rather than ultrasound and computed tomography to exclude synchronous neoplasias often associated with mucoceles and to plan the optimum surgical strategy. The association between appendiceal mucoceles and other neoplasias is relatively frequent, especially with colorectal cancer. Oncogenic activation in the *PIK3CA*-depending pathway may contribute substantially to the pathogenesis of the different solid tumors in the same patient.

## Introduction

The first description of mucoceles of the appendix was made by Rokitansky in 1842, who described a dilatation of the appendiceal lumen due to mucus accumulation [1]. Mucoceles are rare tumors accounting for 8% to 10% of appendiceal tumors and cause 0.2% to 0.4% of all appendectomies [2,3]. Mucoceles are more common in males than in females and affect individuals >50 years more frequently [4].

Mucoceles are distinguished histopathologically as retention cysts (18%), focal or diffuse mucosal hyperplasia (20%), mucinous cystadenomas (52%), or mucinous cystadenocarcinomas (10%) [5]. The natural history, surgical management, and prognosis of appendiceal mucoceles depend on their histological features.

Several neoplasias are associated with appendiceal mucoceles. The most common is adenocarcinoma of the colon and rectum, which is detected in approximately 20% of cases [6-8].

The mitogen-activated protein kinase (MAPK) pathway (including the cascade of RAS, BRAF, MEK1/2, and ERK1/2 proteins) has emerged as a major signaling cascade involved in the control of cell growth, proliferation, and migration in the majority of cancers. A high amount of reports has been published revealing that the *PIK3CA* gene may be somatically mutated in several types of human cancer [9]. In particular, *PIK3CA* is an effector of the phosphatase and tensin homolog (PTEN)–AKT pathway, which is involved in the inhibition of focal adhesion formation, cell spreading and migration as well as in the inhibition of growth factor-stimulated MAPK signaling (alterations in the RAS–BRAF pathway are frequently associated with PTEN-PIK3CA impairments) [10].

In this report, we present the case of a patient affected by adenocarcinoma of the colon and rectum, a bladder carcinoma, and a concomitant appendiceal cystadenoma and the results of the molecular study of the most relevant mutational pathways involved in these tumors.

## Case presentation

A 68-year-old Italian man was admitted to our unit complaining of macrohematuria, rectorrhagia, and rectal tenesmus for about 2 months. No personal or family history of colorectal or urothelial cancer was evidenced. A physical examination revealed pain in the hypogastrium and the right iliac fossa. Laboratory tests showed an increased carcinoembryonic antigen level of 6.46ng/mL (normal range, 0 to 5ng/mL) and increased tissue polypeptide antigen (105U/L; normal range, 0 to 75U/L).

A colonoscopy showed the presence of a rectal lesion at 11cm from the anal margin, which was ulcerated, easily bled, and narrowed the colic lumen. Multiple biopsies were performed and a diagnosis of moderately differentiated adenocarcinoma was made.

Abdominal ultrasonography (US) performed subsequently to stage the rectal cancer showed the presence of two round nodules, measuring 33mm and 30mm, at the anterior aspect of the iliopsoas muscle. Moreover, US revealed a budding lesion (maximum diameter, 24.9mm) affecting the bladder. The patient underwent transurethral cystoscopy and a biopsy of the lesion in the right retrotrigonal region; the diagnosis was grade II urothelial carcinoma.

Furthermore, the patient underwent a total body computed tomography (CT) scan, which showed the rectal tumor and the two abdominal nodules detected previously by US. These nodules were interpreted as swollen lymph nodes, probably of neoplastic origin.

The patient underwent an open anterior rectal resection with a termino-terminal anastomosis and a loco-regional lymphadenectomy. An enlarged appendix and a voluminous whitish soft-tissue lesion requiring an appendicectomy were detected perioperatively. The patient also underwent transurethral resection of the bladder lesion. The postoperative course was uneventful, and the patient was discharged 11 days after surgery.

**Figure 1:**
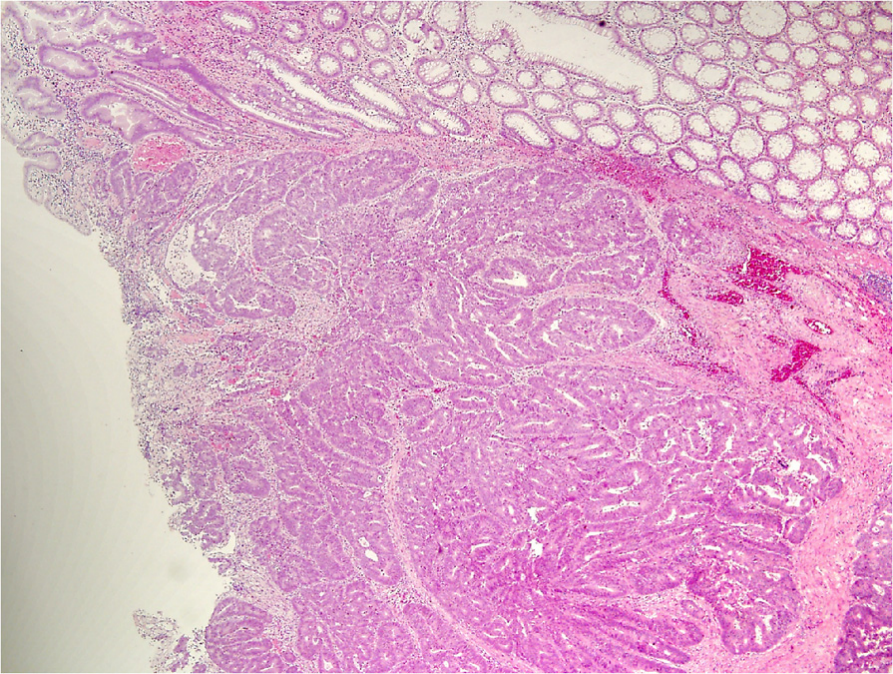
Hematoxylin and eosin stained section of the colonic carcinoma.

**Figure 2:**
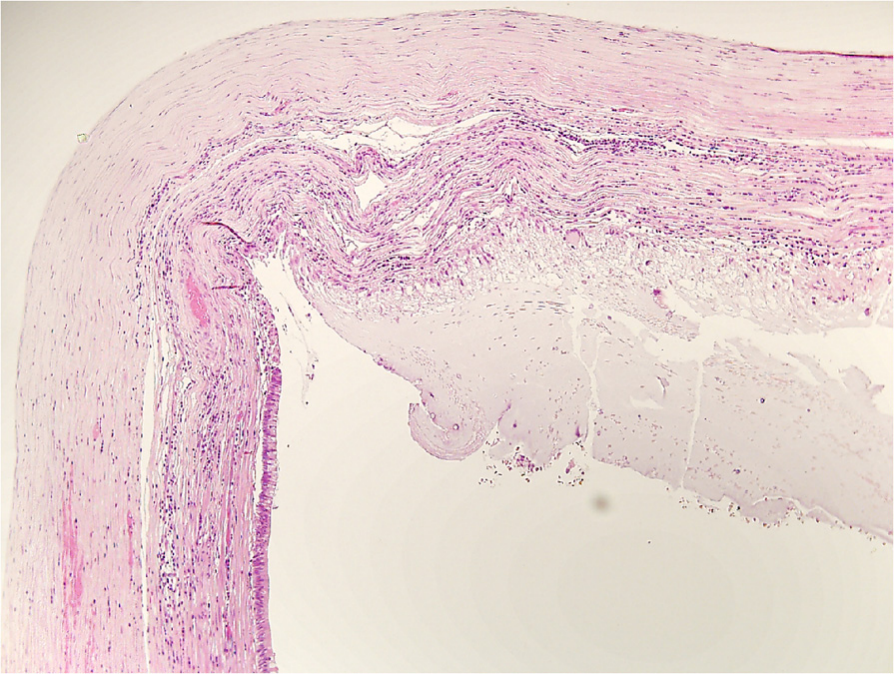
Hematoxylin and eosin stained section of the appendiceal cystadenoma.

**Figure 3:**
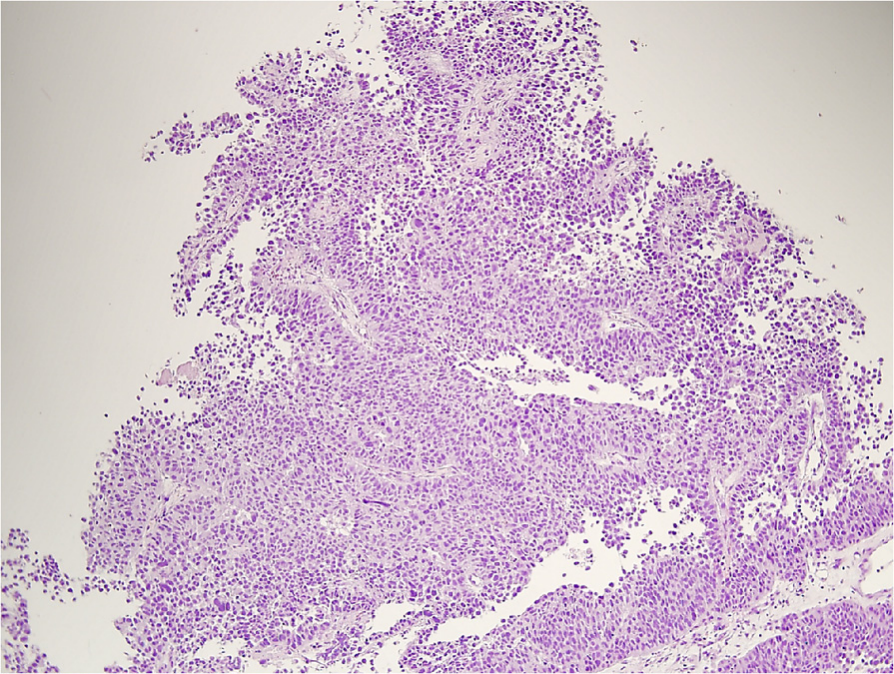
Hematoxylin and eosin stained section of the urothelial carcinoma.

The histological examination of the specimens confirmed the presence of an ulcerated rectal adenocarcinoma, moderately differentiated and completely infiltrating the rectal wall, but not the perirectal fatty tissue (Figure 1). No neoplastic invasion was detected in any of the 27 lymph nodes removed (T3, N0, M0; TNM Stage IIa). Furthermore, it was evidenced that the nodular lesions in the appendix detected preoperatively by imaging were due to accumulation of mucus in the appendiceal lumen caused by a lesion composed of mucus-secreting pseudostratified epithelium, with hyperchromic nuclei and rare pseudo-papillae, diagnosed as a cystadenoma (Figure 2). Finally, a grade 2 urothelial carcinoma infiltrating the subepithelial connective tissue of the bladder was observed (Figure 3). In all cases the resection margins were free of neoplastic invasion.

For mutation analysis, genomic deoxyribonucleic acid (DNA) was isolated from tumor tissue samples, using standard methods. Tumor tissues were estimated to contain at least 70% neoplastic cells by light microscopy. The complete coding sequences and intron–exon boundaries of the *KRAS* exons 2 to 3, *BRAF* exon 15, and the *PIK3CA* exons 9 to 20 were screened for mutations by direct sequencing, using an automated fluorescence-based cycle sequencer (ABIPRISM3100, Applied Biosystems, Foster City, United States of America). Primer sequences were as reported in the Genome DataBase.

**Figure 4:**
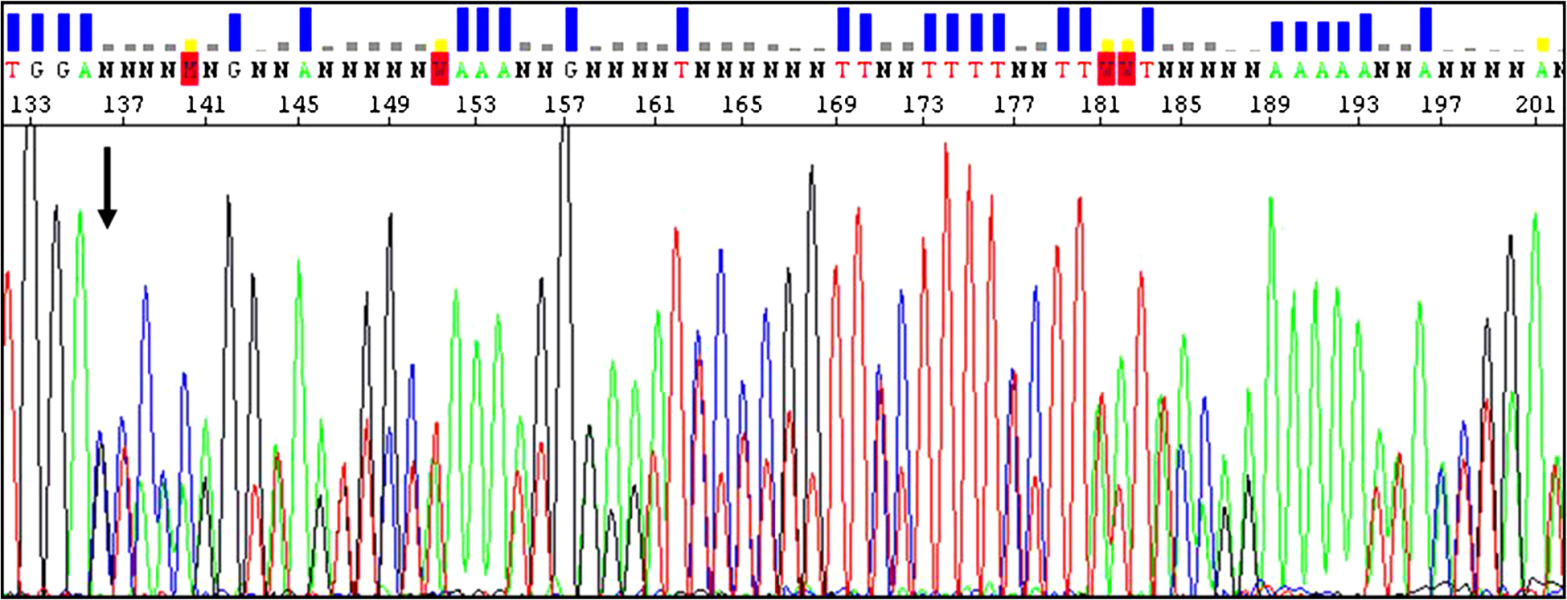
**Sequencing result for the identified *PIK3CA *somatic mutation.** Electropherogram shows the nucleotide sequence of the genomic deoxyribonucleic acid (DNA) from an exemplificative positive sample; arrow indicates the mutation position start within the sequence.

No mutation was detected in both the *KRAS* and *BRAF* genes. For *PIK3CA* mutations, screening revealed that all three samples analyzed carried a variant (p.S553fs*7 in exon 9) which has been firstly reported as associated with the *CRC* (catabolite repression control) gene, although it has been previously described in association with an hematopoietic neoplastic disease [11]. Figure 4 shows the nucleotide sequences for the somatic mutation identified in the *PIK3CA* gene in our patient. The *PIK3CA* sequence variation identified was not present in normal adjacent tissues, indicating that these variants are tumor specific and somatically acquired mutations.

## Discussion

Mucoceles of the appendix are rare tumors, detected in 0.2% to 0.4% of all appendicectomy specimens [2,3]. They are most common in males >50 years old [4].

Mucoceles can be symptomatic or totally asymptomatic and diagnosed accidentally. Symptomatic lesions tend to be associated with malignant forms. The most common signs and symptoms are right-sided acute or chronic abdominal pain (65%) and a palpable abdominal mass (15%). Enterorrhagia due to cecal invagination or ileal volvulus, peritonitis by perforation, sepsis, and urological complications are rarer. In our case, the symptoms were pain at the right iliac fossa, rectorrhagia, and macrohematuria [12].

Abdominal US and CT scan are commonly used to diagnose these neoplasms. A mucocele is usually encapsulated on US, with variable echogenicity in relation to the quantity and fluidity of the mucous contained. The inner wall can appear irregular due to the presence of debris or epithelial hyperplasia. US may be decisive for the differential diagnosis of appendiceal mucocele and acute appendicitis; appendicular diameters of ≥15mm are the threshold for diagnosing appendiceal mucocele, with a sensitivity of 83% and specificity of 92% [13].

The typical CT scan appearance of an appendiceal mucocele is that of a cystic mass with regular walls that demonstrate contrast enhancement and low attenuation content. Parietal calcifications may occasionally be present. Moreover, Kim *et al*. speculated that nodular lesions in a cystic mass with the above-mentioned features suggest a diagnosis of cystadenocarcinoma [14]. US and CT scans are of limited specificity for a differential diagnosis with other neoplasias of the appendix, benign or malignant, and other diseases such as carcinoid tumors, lymphadenopathies, lymphomas, mesenteric cysts, and ovarian masses. Furthermore, a transparietal biopsy of a mucocele is not recommended to reduce the risk of mucous dissemination and avoid pseudomyxoma peritonei [15].

Endoscopic US seems to result in better evaluation of features suspicious for malignancy, even if it is not commonly used [16]. Colonoscopy may be useful for diagnosing appendiceal mucoceles because it offers the possibility of detecting a protrusion of the appendiceal orifice or the presence of yellowish viscous liquid. Furthermore, a colonoscopy is essential for diagnosing synchronous and/or metachronous colic tumors [17]. In our case, the endoscopic examination revealed adenocarcinoma of the rectum, but there was no evidence of mucoceles.

Mucoceles are a heterogeneous group comprising various histopathological lesions, including retention cysts, mucosal hyperplasia, cystadenomas, and cystadenocarcinomas [5]. Appendiceal mucoceles appear as white or reddish smooth-walled cysts. Retention cysts are microscopically delimited by a flat epithelium, dystrophic mi-neralization, fibrosis, and the presence of mucus in the cystic lumen. Mucosal hyperplasia is characterized by epithelial hyperplasia. Cystadenomas are characterized by papillary and glandular proliferation with cellular atypia. Local invasion and peritoneal dissemination are typical features of cystadenocarcinoma. A histopathological classification is critical because it is strictly related to the prognosis.

The treatment of appendiceal mucoceles is essentially surgical, and retention cysts, mucosal hyperplasia, and cystadenomas must be treated with a simple appendicectomy. Larger ileocolic resections, such as a right hemicolectomy, are recommended for cystadenocarcinomas or in the presence of peritoneal pseudomyxoma. This last condition, defined as gelatinous ascites with peritoneal dissemination of neoplastic epithelial cells secreting mucus, is frequently associated with cystadenocarcinoma of the appendix or ovarian cystic tumors and heavily influences prognosis (5-year survival rate, 25%). Aggressive surgical management and intraperitoneal and systemic chemotherapy is recommended for these cases [18,19]. In our case, the presence of disease-free resection margins and the absence of spilled mucus in the peritoneum limited our intervention to a simple appendectomy.

Tissues must be handled with care during surgery to reduce the risk of rupture or peritoneal dissemination [20]. Thus, conventional surgery is preferred over a laparoscopic approach. Gonzáles Moreno *et al*. suggested conversion from laparoscopy to open surgery when the presence of a mucocele is confirmed [21]. Nevertheless, some authors still recommend use of laparoscopy in selected cases.

The association between appendiceal mucoceles and other neoplasias such as gastrointestinal, ovarian, mammary, and renal tumors has been reported. Appendiceal mucoceles and colorectal cancer are associated in approximately 20% of cases [6-8]. Our study provides clear indication that mutational activation of the *PIK3CA* gene participated in tumorigenesis of the three different somatic lesions in our patient. Conversely, no pathogenetic implication was inferred for the *BRAF* and *KRAS* genes, which are members of the MAP kinase (MAPK: RAS-BRAF-MEK-ERK) pathway and mediate cellular response to growth signals. In general, the MAPK and the PIK3CA signaling pathways both play a key role in cell proliferation and survival; in our case, an oncogenic activation occurred in the PIK3CA-dependent pathway, contributing to pathogenesis of the different solid tumors in the same patient.

The prognosis for appendiceal mucoceles depends on the histology and the eventual spillage of mucus into the peritoneum. The 5-year survival rate is 100% for low-grade mucinous neoplasias confined to the appendix; however, it decreases to 45% when peritoneal dissemination occurs [22].

## Conclusions

Appendiceal mucoceles are rare but require adequate surgical treatment, given their malignant potential and the possibility of causing peritoneal pseudomyxoma. It is essential to make a correct preoperative evaluation based on a colonoscopy rather than US and CT to exclude synchronous neoplasias often associated with mucoceles and to plan the optimum surgical strategy. The association between appendiceal mucoceles and other neoplasias is relatively frequent, especially with colorectal cancer. Oncogenic activation in the PIK3CA-dependent pathway may contribute substantially to pathogenesis of the different solid tumors in the same patient.

## Consent

Written informed consent was obtained from the patient for publication of this case report and accompanying images. A copy of the written consent is available for review by the Editor-in-Chief of this journal.

## Competing interests

The authors declare that they do not have any competing interests.

## Authors’ contributions

FP and PP were major contributors in writing the manuscript; AC performed the histological examination and description of the specimens, GP and MC performed the molecular analysis described, and FS and MT performed the surgical operation and final revision of the manuscript. All authors read and approved the final manuscript.
